# Effect of Breastmilk Microbiota and Sialylated Oligosaccharides on the Colonization of Infant Gut Microbial Community and Fecal Metabolome

**DOI:** 10.3390/metabo12111136

**Published:** 2022-11-18

**Authors:** Juan Ding, Runze Ouyang, Sijia Zheng, Yanfeng Wang, Yan Huang, Xiao Ma, Yuxin Zou, Rong Chen, Zhihong Zhuo, Zhen Li, Qi Xin, Lina Zhou, Surong Mei, Jingyu Yan, Xin Lu, Zhigang Ren, Xinyu Liu, Guowang Xu

**Affiliations:** 1 Department of Quality Control, The First Affiliated Hospital of Zhengzhou University, Zhengzhou 450052, China; 2 CAS Key Laboratory of Separation Science for Analytical Chemistry, Dalian Institute of Chemical Physics, Chinese Academy of Sciences, Dalian 116023, China; 3 University of Chinese Academy of Sciences, Beijing 100049, China; 4 Liaoning Province Key Laboratory of Metabolomics, Dalian 116023, China; 5 Department of Nursing, The First Affiliated Hospital of Zhengzhou University, Zhengzhou 450052, China; 6 Liaocheng People’s Hospital, Liaocheng 252000, China; 7 Dalian Municipal Women and Children’s Medical Center (Group), Dalian 116011, China; 8 Department of Pediatric, The First Affiliated Hospital of Zhengzhou University, Zhengzhou 450052, China; 9 Department of Interventional Radiology, The First Affiliated Hospital of Zhengzhou University, Zhengzhou 450052, China; 10 Academy of Medical Sciences, Zhengzhou University, Zhengzhou 450052, China; 11 State Key Laboratory of Environment Health (Incubation), Key Laboratory of Environment and Health, Ministry of Education, Key Laboratory of Environment and Health (Wuhan), Ministry of Environmental Protection, School of Public Health, Tongji Medical College, Huazhong University of Science and Technology, Wuhan 430030, China; 12 Department of Infectious Diseases, The First Affiliated Hospital of Zhengzhou University, Zhengzhou 450052, China

**Keywords:** breastmilk microbiota, breastmilk sialylated oligosaccharides, newborn gut microbiota, fecal metabolome, *Bacteroides*, breastfeeding

## Abstract

The complex microbiota and sialylated oligosaccharides in breastmilk are important bioactive components that affect the gut microbiota. However, the effect of breastmilk microbiota and sialylated oligosaccharides on the gut microbiota during the neonatal period has been largely overlooked. Here, 16S rRNA gene sequencing and metabolomics analysis were applied to the breastmilk and feces of 69 newborns to clarify the link between breastmilk components and the newborn gut. Results showed that *Staphylococcus*, *Enterococcus*, and *Bacteroides* were commonly shared and positively correlated between breastmilk and the neonatal intestine and they were the main bacteria of breastmilk that interacted with the newborn fecal metabolome. Breastmilk *Staphylococcus* mainly interacted with amino acids, whereas *Bacteroides* was involved in the tryptophan, nucleotide, and vitamin metabolism. Breastmilk sialylated oligosaccharides were related to *Bacteroides* and amino acids of the newborn fecal metabolites. Moreover, *Bacteroides* was related to the interaction between breastmilk 3′-sialyllactose and newborn fecal metabolites in the mediation effect models. Finally, we pointed out that breastmilk *Bacteroides* was important in the milk–gut interaction, and it was negatively associated with waist circumference in infants aged 1 year. Our study provides a scientific basis for understanding the role of breastmilk in the development of newborn gut microbiota and metabolome.

## 1. Introduction

Exclusive breastfeeding is recommended during the first six months of life (http://www.who.int/nutrition/topics/exclusive_breastfeeding/en/ (accessed on 15 November 2022)). However, for newborns who cannot be breastfed, specifically designed formula milk is required, and continuous efforts are made to improve the ingredients of formula milk to make it more similar to breastmilk. Understanding the role of breastfeeding in newborns is supposed to be meaningful to this endeavor. One of the most important roles of breastfeeding is to shape the neonatal gut microbiota, thus having a far-reaching impact on health [1,2]. It is believed that the effect of breastmilk on the early gut microbiota is, at least partially, modulated by specific bioactive compounds (i.e., microbiota and sialylated oligosaccharides) contained in breastmilk [3,4].

The breastmilk microbiota refers to a complex community of bacteria harbored in breastmilk. The identification of many commonly shared bacteria between breastmilk and the infant gut indicated that breastmilk could potentially transfer certain bacteria into the infant intestine [5,6,7], although maternal skin, family members, and environment are all also potential sources of infant gut microbiota [8,9,10]. Breastmilk sialylated oligosaccharides are especially important human milk oligosaccharides, with the function of promoting the growth of gut microbiota, protecting against infectious diseases, and aiding brain development [11,12,13]. 3′-Sialyllactose (3′-SL) and 6′-sialyllactose (6′-SL) are the main forms of sialylated oligosaccharides [14]. Moreover, breastmilk 3′-SL and 6′-SL have been demonstrated to be the most widely bacteria-associated sialylated oligosaccharides [7,15].

Until now, interactions between breastmilk microbiota and sialylated oligosaccharides and the infant gut have been confirmed in many large population cohorts, such as the CHILD cohort and the SKOT III cohort [6,7]. However, most of these studies mainly focused on mid- and late lactation (one month after birth) [7,16,17,18,19], rather than the first week of life. Due to the high variation in breastmilk microbiota and sialylated oligosaccharides across lactation durations [5,20,21], the milk–gut interaction during mid- and late lactation may not comprehensively reflect the role of breastmilk bacteria or sialylated oligosaccharides in the development of the newborn gut microbiota. Additionally, as the essential residue of sialylated oligosaccharides, the relationship between sialic acid (SA) and gut microbiota has not received enough attention.

The fecal metabolome consists of numerous gut microbiota–host cometabolites, and its analysis could provide supplementary functional readout information on the gut microbiota [22]. Most recent studies of breastmilk on shaping the infant gut microbiota have mainly focused on the variation in gut microbial composition [5,6], while the metabolic function of microbiota has been largely ignored. It has been reported that diet (namely breastmilk or formula milk) could cause changes in infant fecal metabolism in early life [23,24]. However, the specific role of breastmilk microbiota and sialylated oligosaccharides in affecting the newborn fecal metabolome is poorly understood.

To identify the potential effect of breastmilk bioactivate components on the establishment of the newborn gut microbiota, 16S rRNA gene sequencing and metabolomics approaches were used to assess the association of the breastmilk microbiota and sialylated oligosaccharides with the neonatal gut microbiota and fecal metabolome. Furthermore, the main bacteria involved in the milk–gut interaction were sought, and their role in infant growth was explored.

## 2. Materials and Methods

### 2.1. Study Design and Sample Collection

Chinese mothers aged 20–40 years old were enrolled after they delivered a healthy and full-term infant in the First Affiliated Hospital of Zhengzhou University (Zhengzhou, China) between April and December 2017 (n = 69). The basic health status of the mothers and infants at birth was recorded by the standardized questionnaire, which is summarized in Table S1 and Table 1. All the newborns were born at full term and there was an equal gender distribution. Moreover, 35 of the 69 infants were naturally delivered and 16 of the 69 newborns were fed only with breastmilk at the time of sample collection. No newborns were treated with antibiotics, and 31 mothers were exposed to antibiotics after delivery. Infant waist circumference was obtained during the medical examination at 1 year old. Specifically, the waist circumference was measured in duplicate just above the superior iliac crests, with the infant lying down, and the mean waist circumference was calculated. All the mothers provided informed consent.

All breastmilk samples were collected around the first week after delivery. Breastmilk samples were collected 2 h after breastfeeding according to standard protocol. Furthermore, 10 mL breastmilk samples were collected manually in a sterile tube after the mothers cleaned their hands, nipples, and surrounding area with sterile saline swabs twice, discarding the first drops. The newborn fecal samples were taken within 24 h of breastmilk collection. Briefly, 200–500 mg infant fecal samples were scraped with a spoon from diapers into a sterile stool container (Sarstedt 80.734.001, Nümbrecht, Germany) within 24 h of breastmilk collection. All the samples were kept frozen at −20 °C for no more than 24 h and then stored at −80 °C for further analysis.

### 2.2. DNA Extraction and Sequencing

The genomic DNA of feces and breastmilk samples was extracted based on a previously described method [25], with slight modifications to the lysis process of breastmilk samples. Briefly, 1 mL of breastmilk was thawed on ice and centrifuged at 8600× *g* for 20 min at 4 °C. The pellet was resuspended with 0.8 mL lysis buffer and transferred to a 2 mL Matrix E tube (MP Bio, Irvine, CA, USA). A FastPrep 24 Instrument (MP Bio, Irvine, CA, USA) was used for mechanical lysis of the cells at 5.5 m/s for 45 s. Then cells were enzymatically lysed with 50 µL Mut-Lys Mix (100 mg/mL lysozyme, 5 U/µL mutanolysin) at 37 °C for 30 min. Then the lysate of breastmilk samples was used for DNA extraction.

The V4 region of the 16S rRNA gene was amplified with 515F/806R primers [26] and sequenced following a previous study [25] with slight modifications. Briefly, the PCR system was set as follows: 2× Phusion Master Mix with GC buffer (for breastmilk samples, Thermo Scientific, Graciuno, Vilnius, Lithuania) or 2.5× Five Prime Hot Master Mix (for fecal samples, Thermo Scientific, Graciuno, Vilnius, Lithuania), 200 nM of each primer, and 20 ng of the genomic DNA template. Illumina MiSeq platform (RTA version 1.18.54, MSC version 2.4, Illumina, San Diago, CA, USA) with the MiSeq Reagent Kit V2 (Illumina, San Diago, CA, USA) was used for sequencing 250 bp paired-end of the amplified V4 region of 16S rRNA gene.

### 2.3. Microbial Data Pre-Processing

Quantitative Insights into Microbial Ecology 2 (QIIME 2) software V2021.2 was used to analyze the sequencing data [27]. Firstly, demultiplexed fastq files were imported into QIIME 2 with the manifest importing tool. Next, DADA2 was used for quality control, paired-end merging, and chimera filtering of the paired-end reads [28]. Then, breastmilk and fecal samples with less than 1000 features were removed from the follow-up analysis, which led to 62 breastmilk and 57 newborn feces being kept. In summary, a total of 1,610,253 and 2,772,451 high-quality sequencing reads were obtained for breastmilk and the neonatal gut, respectively, which resulted in a mean of 25,972 ± 13,879 sequencing reads per breastmilk sample and 48,639 ± 28,208 reads per fecal sample.

The SILVA 138 database was used for the taxonomy identification of unique amplicon sequence variants (ASVs) at 99% similarity. After filtering ASVs that existed in less than 10% of breastmilk or newborn feces [29], 153 and 45 unique ASVs were assigned in breastmilk and newborn feces, respectively. The taxonomic composition table of samples was exported at the genus and ASV levels, and the relative abundances of the taxa at genus and ASV levels were calculated by total sum scaling.

### 2.4. Sialic Acid and Sialylated Oligosaccharides Quantification

SA, 3′-SL, and 6′-SL were quantified on an online solid-phase extraction-hydrophilic interaction chromatography (SPE-HILIC-MS) platform using a previously described method with slight modifications [30]. Briefly, 200 μL of breastmilk was centrifuged at 8600× *g* for 20 min at 4 °C to remove the lipid content. Then 200 μL of ethanol with an internal standard (SA-^13^C6) was added to the skim milk before centrifugation at 15,000× *g* for 15 min at 4 °C to remove the proteins. Finally, 50 μL of the supernatant was freeze-dried and redissolved in 200 μL of the acetonitrile (ACN)/H_2_O *(v*/*v* = 1/1) solvent for analysis. The parameters of the online SPE-HILIC-MS platform and quantification of sialic acid and sialylated oligosaccharides are described in the supplementary materials.

### 2.5. Nontargeted Neonatal Fecal Metabolomic Analysis

Approximately 50 mg of fecal samples and 1 mL of MeOH/H_2_O (*v*/*v* = 1/1) with internal standards (ISs, Table S2) were mixed and then homogenized twice (30 Hz, 1 min) in a mixed ball grinding machine (MM400, Retsch Technology, Han, Germany). The mixture was centrifuged at 4 °C, 10,000× *g* for 10 min, then 800 μL of the supernatant was collected for lyophilization. The freeze-dried samples were resuspended with 300 μL of ACN/H_2_O (*v*/*v* = 1/3) on a vortex mixer and the mixture was centrifuged at 4 °C, 10,000× *g* for 10 min. Then the supernatant was filtered to remove impurities with a 0.22 μm filter membrane and then used for later analysis. The detailed method for liquid chromatography-mass spectrometry (LC-MS)-based nontargeted metabolomic analysis and metabolite identification is described in the supplementary materials.

### 2.6. Statistical Analysis

The “core-metrics-phylogenetic” methods in QIIME 2 were applied to assess the α- and β- diversity indexes. The α-diversity was calculated by observed features, and the β- diversity was calculated with the Jaccard distance and visualized by a three-dimensional principal coordinate analysis (PCoA) in QIIME 2. Stratification of the breastmilk microbiota was performed by partitioning around the medoid method based on Jensen–Shannon divergence (PAM-JSD) [31]. The Calinski–Harabasz index (CH index) and obs.silhouette were combined to evaluate the optimal cluster number [32]. The cluster, MASS, clusterSim, and ade4 packages in R studio (version 4.0) were used for the microbial clustering.

Partial correlation analysis was conducted to evaluate the influence of breastmilk microbiota and sialylated oligosaccharides on the infant gut microbiota and fecal metabolome by SPSS 25.0 (adjusting for feeding patterns), and coefficients with |R_1_| > 0.2 and *p* < 0.05 were considered significant. Spearman correlation coefficients were calculated to assess the association between the breastmilk microbiota and sialylated oligosaccharides and the association between the infant gut microbiota and fecal metabolites within GraphPad Prism 9.0, and coefficients with |R_2_| > 0.2 and *p* < 0.05 were considered significant. Power analysis of the correlation coefficients was calculated by the pwr package in R studio to estimate the reliability of the associations. With a correlation coefficient of 0.3 and a significance level of 0.05, the study power is 60%.

Mediation effect analysis was conducted by stepwise linear regression. Briefly, breastmilk sialylated oligosaccharides, bacteria, and fecal metabolites were set as independent variables (X), intermediate variables (M), and dependent variables (Y), respectively. Associations between X and M, M and Y, and X and Y were calculated, respectively. If all the above associations were significant, then the mediating effect was significant. A binary logistic analysis was used to evaluate the influence of neonatal *Bacteroides* and its associated fecal metabolites on the maternal and infant waist circumference at the age of 1 year.

All the taxa-bar plots, box plots, pie charts, and heatmaps were generated by GraphPad Prism 9.0, and Cytoscape (version 3.7.1) was applied to visualize the correlation network.

## 3. Results

### 3.1. Correlation of Breastmilk Bacteria with the Newborn Gut Microbiota

**Figure 1:**
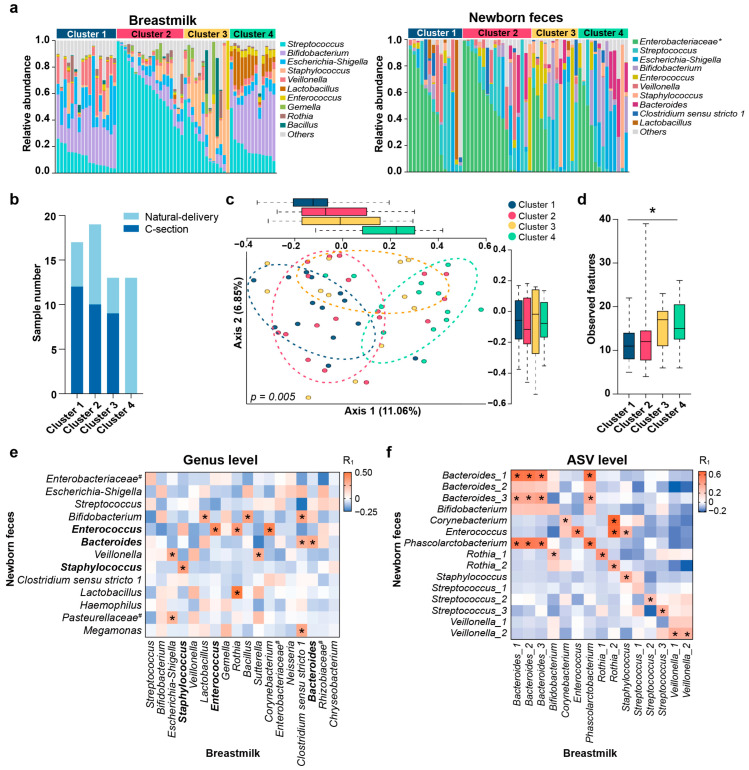
Breastmilk bacteria are correlated with newborn gut microbiota in the first week of lactation. (**a**) Taxa-bar plot of mean relative abundance of neonatal breastmilk microbiota and gut microbiota at the genus level. Top 10 genera in breastmilk or fecal samples are included. Bacteria other than top 10 are grouped as ‘Others’. (**b**) Number of newborns based on delivery mode in the four breastmilk microbial clusters. (**c**) PCoA plot based on Jaccard distance of the neonatal gut microbiota corresponding to the four breastmilk microbial clusters. (**d**) Boxplots of alpha diversity calculated by observed features of the neonatal gut microbiota in the four breastmilk microbial clusters (Mann–Whitney U test followed by Bonferroni correction, * *p* < 0.05); (**e**,**f**) Heatmap of partial correlation analysis between breastmilk microbiota and newborn gut microbiota at the genus level (**e**) and the ASV level (**f**). Bacteria with relative abundance >1% are included. Asterisks (*) in heatmap mean significant correlation (|R_1_| > 0.2 and *p* < 0.05). Pound sign (#) indicates the unclassified bacteria at the genus level. Genera in the abscissa are members of breastmilk, and genera in the ordinate are the bacteria of newborn gut microbiota.

The neonatal breastmilk and gut microbial composition were first characterized separately. At the genus level, the neonatal breastmilk microbiota was dominated by *Streptococcus*, *Bifidobacterium*, *Escherichia-Shigella*, *Staphylococcus*, and *Veillonella*, while *an unclassified Enterobacteriaceae*, *Escherichia-Shigella, Streptococcus,* and *Bifidobacterium* were the most prevalent bacteria in newborn feces (Figure 1a). In addition, several well-known neonatal gut bacteria, such as *Lactobacillus*, *Enterococcus*, and *Bacteroides*, occupied a large proportion of the breastmilk microbiota during early lactation (Table S3). Cluster analysis based on the PAM-JSD method revealed that the neonatal breastmilk microbiota could form four clusters in terms of the Calinski–Harabasz (CH) index (Figure 1a and Figure S1a,b). The delivery mode might be a factor in the stratification of breastmilk microbiota (Figure 1b). Notably, breastmilk microbiota clusters were related to the distinct diversity of the neonatal gut microbiota (Figure 1c,d), that is, the gut microbiota of newborns belonging to Cluster 4 pretended to have a higher alpha diversity than the other three clusters. Additionally, the delivery mode and maternal antibiotic usage were also impact factors of the neonatal gut microbiota (Figure S1c–e).

The comparison of the taxa composition at the genus level between breastmilk and newborn feces revealed that the dominant genera in neonatal feces were generally similar to those in breastmilk but with distinct relative abundances (Figure S1f and Tables S3 and S4). For instance, *Escherichia-Shigella*, *Streptococcus*, and *Bifidobacterium* were all dominant genera in breastmilk and newborn feces. Further correlation analysis showed that most of the breastmilk microbiota were positively correlated with the newborn gut microbiota, suggesting that breastmilk bacteria likely promoted the colonization of the newborn gut. Notably, the relative abundances of *Staphylococcus*, *Enterococcus*, and *Bacteroides* in the neonatal gut were positively correlated with those same genera in breastmilk (all R_1_ > 0.3, *p* < 0.05 and power > 0.6) (Figure 1e). Additionally, an intense correlation was observed between breastmilk *Rothia* and *Lactobacillus* of the neonatal gut (R_1_ > 0.5, *p* < 0.05 and power > 0.8).

Furthermore, the shared individual ASVs between breastmilk and newborn feces were assessed. A median of eight ASVs was shared with the actual mother–newborn dyads at the first week of life (range: 2–15). Most of the shared ASVs belonged to *Bacteroides* (six ASVs), *Bifidobacterium* (three ASVs), *Rothia* (five ASVs), *Streptococcus* (five ASVs), and *Veillonella* (four ASVs). Many strongly correlated milk–gut ASV pairs were observed when we further assessed the correlations between relative abundances of co-occurring ASVs in breastmilk and the infant gut. For instance, two *Bacteroides* ASVs, two *Rothia* ASVs, and an *Enterococcus* ASV appeared to have significantly positive associations between milk and gut (all R_1_ > 0.3, *p* < 0.05 and power > 0.8) (Figure 1f).

### 3.2. Interaction of the Milk–Gut Co-Occurring Genera in Breastmilk with the Overall Neonatal Fecal Metabolome

The fecal metabolome was composed of a large number of microbial metabolites, which could reflect the metabolic function of gut microbiota. Therefore, the crosstalk between the breastmilk microbiota and the newborn fecal metabolome was further analyzed. Among the 134 neonatal fecal metabolites identified in the study (Table S5), amino acids were the most diversified fecal metabolites and xenobiotics were the most breastmilk bacteria-associated compounds in the neonatal fecal metabolome (Figure S2).

**Figure 2:**
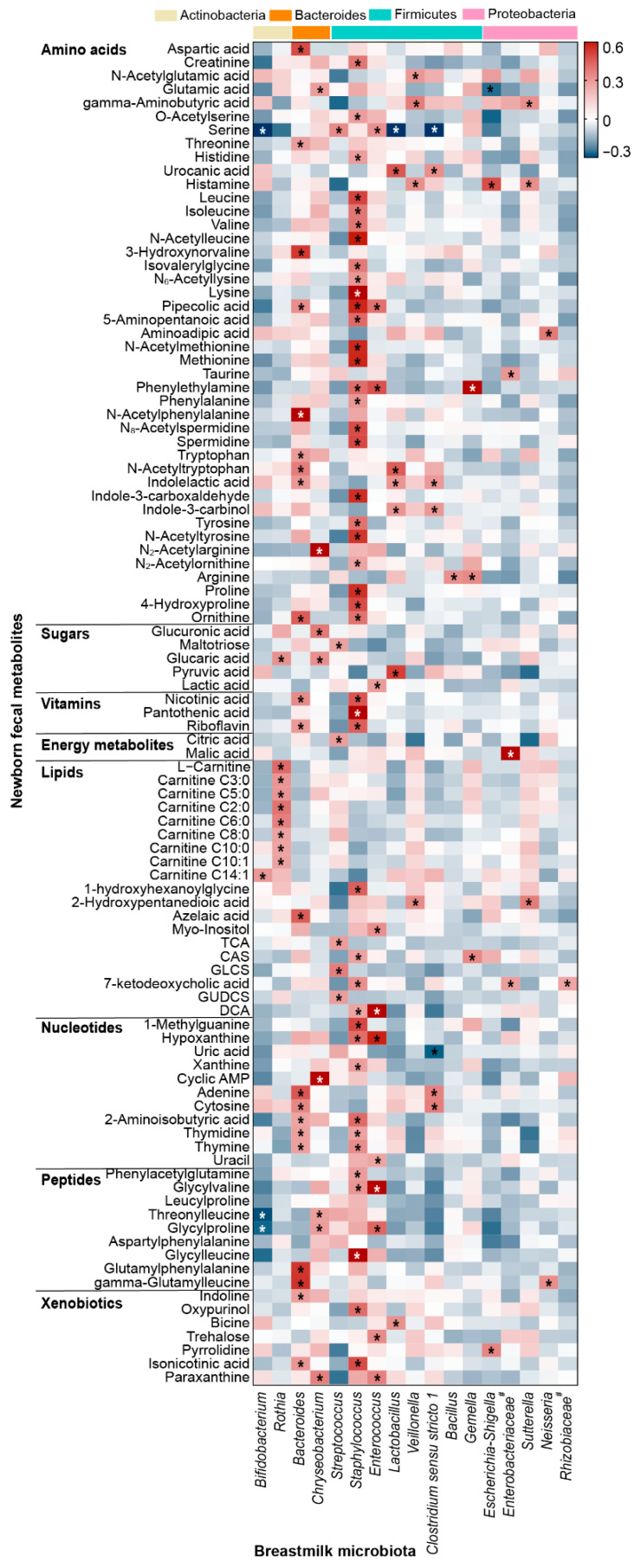
Heatmap of partial correlation analysis between breastmilk microbiota and the neonatal fecal metabolome. Correlations with |R_1_| > 0.2 and *p* < 0.05 are marked with an asterisk (*). Pound sign (#) indicates the unclassified bacteria at the genus level.

In general, more than 70% of the fecal metabolites identified in the study interacted with breastmilk microbiota (Table S6). *Staphylococcus*, *Enterococcus*, and *Bacteroides*, the co-occurring genera between breastmilk and the neonatal gut, played crucial roles in the interaction with fecal metabolites (Figure 2 and Table S6). *Staphylococcus* was associated with the greatest variety and the largest number of fecal metabolites, and most of them belonged to amino acids and their derivatives. For instance, amino acids related to glycine, serine and threonine metabolism, leucine, isoleucine and valine metabolism, lysine metabolism, tyrosine metabolism, and arginine and proline metabolism presented intense interactions with *Staphylococcus* (Figure 2 and Figure S2c). In addition, *Staphylococcus* was related to other basal metabolites of newborns, such as vitamins, nucleotides, and bile acids (Figure 2). *Enterococcus* was the second most widely interacting genus with fecal metabolites. Lactic acid, uracil, and certain secondary metabolites such as glycylvaline and glycylleucine were the main fecal metabolites associated with breastmilk *Enterococcus* (power > 0.51) (Figure 2 and Table S6). Furthermore, deoxycholic acid (DCA), a secondary unconjugated bile acid, had the strongest correlation with *Enterococcus* (R_1_ > 0.6, *p* < 0.05, and power > 0.99) (Table S6). Furthermore, *Bacteroides* mainly interacted with tryptophan metabolites (such as tryptophan, N-acetyltryptophan, and indolelactic acid) and vitamin metabolites (power > 0.59) (Figure 2 and Figure S2c). Additionally, nucleotide metabolites, including adenine, cytosine, thymidine, and thymine, were also significantly correlated with *Bacteroides* (power > 0.55).

### 3.3. Association between Breastmilk Sialylated Oligosaccharides and Fecal Metabolites

Sialylated oligosaccharides were another key bioactive substance in breastmilk that could interact with microbiota both in breastmilk and the infant gut. It has been reported that breastmilk sialylated oligosaccharides were digestible only under the influence of the infant gut microbiota. Additionally, existing research indicated that breastmilk contains higher concentrations of sialylated oligosaccharides than formula milk [33]. 3′-SL and 6′-SL are the main forms of sialylated oligosaccharides, and they could be broken down into SA under the action of the gut microbiota [34]. Therefore, the link between breastmilk SA/3′-SL/6′-SL and newborn fecal metabolites, as well as the role of bacteria in it, were further studied.

**Figure 3:**
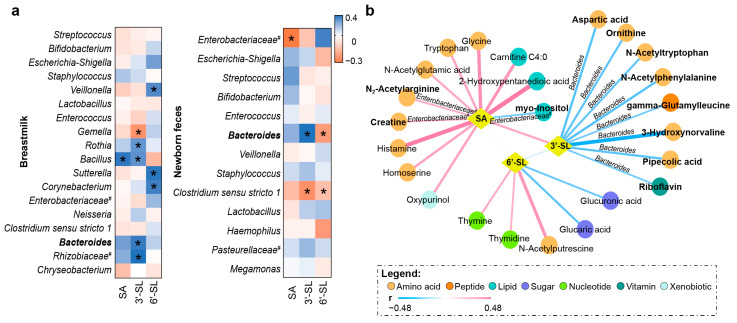
Interaction between breastmilk sialylated oligosaccharides and the newborn gut microbiota as well as fecal metabolome. (**a**) Heatmap of the associations between breastmilk sialylated oligosaccharides and breastmilk microbiota or newborn gut microbiota. Asterisks (*) in heatmap mean significant correlation (|R_1_| > 0.2 and *p* < 0.05). Pound sign (#) indicates the unclassified bacteria at the genus level. (**b**) Partial correlation analysis of breastmilk sialylated oligosaccharides and the newborn fecal metabolites. Correlations with |R_1_| > 0.2 and *p* < 0.05 are included in the network. Fecal metabolites marked in bold indicate the gut bacteria-mediated correlations between breastmilk SA/3′-SL/6′-SL and infant fecal metabolites.

By analyzing the association between the microbiota and breastmilk SA/3′-SL/6′-SL, we found that breastmilk 3′-SL and 6′-SL interacted with more bacteria than SA. Moreover, we found that *Bacteroides* was the only genus that had a similar interaction with 3′-SL in both breastmilk and neonatal feces (Figure 3a). Meanwhile, correlation analysis between breastmilk SA/3′-SL/6′-SL and fecal metabolites suggested that many gut microbial metabolites (i.e., tryptophan, N-acetyltryptophan, and riboflavin) interacted with breastmilk SA/3′-SL/6′-SL (power > 0.56) (Figure 3b), which favors the idea that the utilization of breastmilk SA/3′-SL/6′-SL was related to the gut microbiota. Then mediating effect models further indicated that all the correlations between breastmilk 3′-SL and fecal metabolites were related to gut *Bacteroides*, suggesting that breastmilk 3′-SL might interact with gut microbiota, especially *Bacteroides*, causing changes in the fecal metabolome (Figure 3b and Figure S3 and Table S8).

### 3.4. Relationship of Breastmilk Bacteroides with Infants’ Waist Circumference

In summary, we found that *Bacteroides* was not only strongly correlated in breastmilk and newborn gut but was also linked to the infant tryptophan and vitamin metabolism (Figure S4a). Moreover, *Bacteroides* was also involved in the crosstalk between 3′-SL and fecal metabolites. Therefore, *Bacteroides* was considered the important bacteria in the milk–gut interaction.

**Figure 4:**
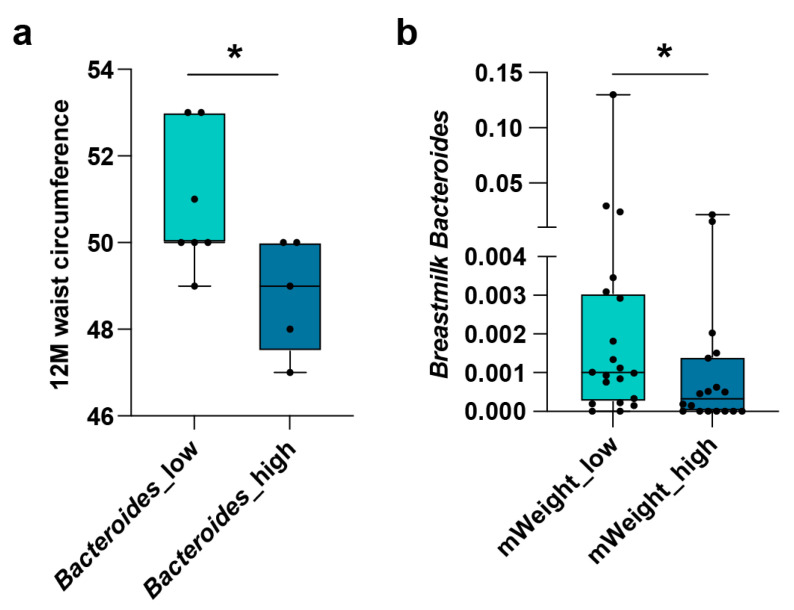
Association of breastmilk *Bacteroides* with infant waist circumference aged 1 year and pre-pregnancy maternal weight. (**a**) Boxplot of infant waist circumference aged 1 year based on tertiles of breastmilk *Bacteroides*. *Bacteroides*_low indicates the lowest tertile and *Bacteroides*_high indicates the highest tertile. (**b**) Boxplot of relative abundance of breastmilk *Bacteroides* based on the median of pre-pregnancy maternal weight. The mWeight_low group includes newborns with pre-pregnancy maternal weight below the median, and the mWeight_high group includes newborns with pre-pregnancy maternal weight above the median (Mann–Whitney U test, * *p* < 0.05).

Waist circumference is a useful indicator of abdominal obesity in infants [35]. Thus, the potential correlation of *Bacteroides* in breastmilk with the waist circumference of the infants at 1 year old was specifically analyzed. We found that breastmilk *Bacteroides* was negatively related to infants’ waist circumference at the age of 1 year (Figure 4a). Then we further sought *Bacteroides*-associated neonatal fecal metabolites that potentially link to infants’ low waist circumference at the age of 1 year (Table S9 and Figure S4b). Linear regression models indicated that amino acids such as N-acetyltyrosine, proline, and 4-hydroxyproline and vitamins including nicotinic acid and riboflavin might be related to the low waist circumstance in infants at the age of 1 year (Table 2 and Figure S4b).

As expected, the pre-pregnancy maternal weight and the waist circumference of infants aged 1 year were positively correlated (r = 0.45). Moreover, breastmilk *Bacteroides* and pre-pregnancy maternal weight were also negatively associated with each other (Figure 4b). Thus, we speculate that breastmilk *Bacteroides* might be a mediator of the mother-to-child metabolic phenotype correlation.

## 4. Discussion

By using multiple analytic approaches, we found the link between breastmilk microbiota and sialylated oligosaccharides with the establishment of the newborn gut microbiota during the first week of life and proposed a possible *Bacteroides*-related mechanism for breastmilk to reduce infant obesity risk later in life.

Breastmilk bacteria contribute to the establishment of infant gut microbiota, especially during the neonatal period. More than 80% of the ASVs in the newborn intestine overlapped with those in breastmilk during the first week of life; however, the ratio dropped to 33% and 23% at the ages of 5 and 9 months [7], suggesting the great contribution of breastmilk microbiota to the establishment of the newborn gut microbiota. Breastmilk could transfer certain bacteria to the infant intestine, and many commonly shared genera in breastmilk and the newborn gut microbiota have been identified [5,6,7,36,37,38]. Similarly, our data also support that the milk–gut interactions of *Staphylococcus*, *Enterococcus,* and *Bacteroides* occur in the first week of life. The co-occurrence of bacteria in breastmilk and the newborn gut indicated that the genera might have the ability to transfer to the gut and contribute to the beneficial health effects of breastfeeding [39].

Many of the co-occurring bacteria in our study are widely considered to be related to the positive health outcomes of infants. For instance, *Rothia* was reported to be protective against atopic wheeze [40] and *Veillonella* could convert lactate to short-chain fatty acids and is therefore associated with positive health and immunoregulatory effects [41,42]. Additionally, some of the co-occurring genera identified in the study have the ability to promote the utilization of human milk oligosaccharides, such as *Staphylococcus*, *Bacteroides*, and *Bifidobacterium* [43,44]. Further mechanistic studies are needed to verify whether and how breastmilk microbiota seed the infant gut and produce health effects because many exogenous factors could also affect both breastmilk microbiota and gut microbiota, such as maternal gut microbiota and environmental bacteria.

As the golden source of nutrition for newborns, breastmilk could provide metabolites necessary for infant growth, such as amino acids [45]. Our study made the first attempt to clarify the potential impact of breastmilk bacteria on newborns from the perspective of the gut metabolome. Breastmilk feeding induces variation in the gut metabolome [23], and our data further supported that breastmilk microbiota widely contributed to this. The commonly shared genera in milk and the gut were the major members interacting with the infant fecal metabolome, suggesting that breastmilk microbiota might influence the fecal metabolome by colonizing the gut. Notably, many of the breastmilk bacteria-associated metabolites have been reported to be related to positive health outcomes. Branched-chain amino acids (BCAAs) are involved in the regulation of energy homeostasis and are crucial in neonatal nutrition [46,47]. The strong association between breastmilk *Staphylococcus* and leucine, isoleucine, and valine metabolism in newborns indicated that breastmilk feeding might promote the BCAA metabolism in newborns. Tryptophan metabolism is involved in the newborn neurodevelopment and the immune system [48], and our data showed that breastmilk *Bacteroides* play a potential role in this process. Meanwhile, it is essential to consider alternative explanations for the correlation analysis between breastmilk microbiota and fecal metabolites. That is, microbial metabolites in breastmilk possibly interact with the gut metabolic pathway. It is also possible that breastmilk microbiota might influence the newborn fecal metabolome in a gut bacteria-independent manner.

Sialylated oligosaccharides are a kind of important breastmilk oligosaccharides that could modify gut microbiota and are linked to beneficial effects on health. Taking 3′-SL and 6′-SL as examples, we showed that sialylated oligosaccharides of neonatal breastmilk were more associated with the gut microbiota compared with those of 5-month breastmilk [7]. However, in contrast to the breastmilk microbiota, breastmilk 3′-SL and 6′-SL interacted with fewer gut bacteria and fecal metabolites. Sialylated oligosaccharides might be more easily metabolized by microbiota, as 3′-SL and 6′-SL interacted with more bacteria than SA. Bacteria was important in the association between breastmilk sialylated oligosaccharides and fecal metabolome. It has been reported that 3′-SL could be metabolized by *Bacteroides* [44,49]. Consistently, we supported that *Bacteroides* was related to the utilization of breastmilk 3′-SL in the infant’s gut, as *Bacteroides* was mediated in the interaction between 3′-SL and the neonatal fecal metabolome. 6′-SL was the most abundant sialylated oligosaccharide in breastmilk [14], and our study indicated its important role in pyrimidine metabolism and the glucuronate pathway. However, genera mediating the interaction between SA/6′-SL and newborn fecal metabolites were not found in this study, which requires further study.

Breastfeeding could reduce the risk of obesity in later infancy [39]. Our study indicated that breastmilk *Bacteroides* potentially contributed to this. *Bacteroides* have been widely reported to reduce the risk of obesity in children [50,51]. Similarly, our study observed a negative correlation between the waist circumference of infants aged 1 year and breastmilk *Bacteroides*. Furthermore, we found that newborn vitamin B complexes, namely, nicotinic acid and riboflavin, might be related to the association between *Bacteroides* and the infant waist circumference. Consistently, it has been reported that vitamin B complexes were involved in energy metabolism and linked with obesity [52,53]. Altogether, our findings revealed a possible *Bacteroides*-related mechanism in breastmilk reducing the risk of childhood obesity.

Breastmilk is the best choice for newborn nutrition. However, when it is not available or insufficient, formula milk is an effective alternative. Our research describes the associations of breastmilk bacteria and sialylated oligosaccharides with the newborn gut microbiota and provides ideas for developing formulas that are functionally similar to breastmilk. Moreover, our study might provide references for developing probiotics and prebiotics to establish a healthy gut microbial community in newborns.

Nevertheless, this study has some limitations. The number of mother–infant pairs included in the analysis was low, and all the correlations identified in the study need to be verified in larger cohorts, especially for the associations with high power values. The high sample loss rate of the infants at the age of 1 year led to only a few infants being evaluated in the correlation analysis between breastmilk *Bacteroides* and waist circumstance. Furthermore, our study lacks a control group to show the gut microbiota composition and the importance of breastmilk microbiota and sialylated oligosaccharides when infants are under formula-only feeding. Moreover, other relevant cofactors affecting the newborn gut microbiota, such as the delivery mode and maternal antibiotic usage, should also be paid attention. 16S rRNA gene sequencing has a limited capacity to identify taxa at the species and strain levels, and further metagenomic or culturomic studies are required.

## 5. Conclusions

Our study provides evidence that breastmilk bioactive components are linked to the newborn gut microbiota assembly and thus affect the fecal metabolome. *Staphylococcus*, *Enterococcus*, and *Bacteroides* co-occurred between breastmilk and neonatal intestine, and extensively interacted with the newborn fecal metabolome. *Bacteroides* was involved in the interaction between breastmilk 3′-SL and newborn fecal metabolites. Altogether, *Bacteroides* was important in breastmilk–gut interactions and might be associated with reducing obesity risk in infants at 1 year old.

## Figures and Tables

**Table 1:** Characteristics of the mothers and infants included in the study.

	Newborns (n = 69)
**Sampling days**	4 (3–5)
**Delivery mode**	
Natural delivery	35
C-section	34
**Maternal BMI (kg/m^2^) ^1^**	
Pre-pregnancy	20.47 (18.79–22.86)
Pre-delivery	26.47 (24.61–29.83)
**Antibiotic usage**	
Maternal intrapartum ^2^	31
Infant antibiotic	0
**Infant sex**	
Male	25
Female	44
**Birth weight (kg)**	3.30 (3.10–3.65)
**Infant waist circumference at 1-year-old (cm) ^3^**	50.00 (48.00–51.00)

^1^ Missing data on 27 newborns; ^2^ Missing data on 9 newborns; ^3^ Missing data on 48 newborns. All the continuous variations are given as median and interquartile ranges.

**Table 2:** Association of fecal metabolites with infant waist aged 1 year.

Fecal Metabolites	B			*p*
	Mean	Lower Limit	Upper Limit	
Proline	−25.341	−50.400	−0.283	0.048
Isonicotinic acid ^1^	−339.222	−611.637	−66.808	0.019
Nicotinic acid ^1^	−713.762	−1285.478	−142.045	0.019
N-Acetyltyrosine	−1172.828	−2272.172	−73.484	0.038
4-Hydroxyproline	−4273.696	−7843.868	−703.524	0.023
2-Aminoisobutyric acid	−11,195.494	−21,012.130	−1378.859	0.029
Riboflavin^1^	−30,704.250	−51,191.441	−10,217.060	0.007

^1^ Breastmilk *Bacteroides*-related fecal metabolites.

## Data Availability

The data for this study have been deposited in the European Nucleotide Archive (ENA) at EMBL-EBI under accession number PRJEB 49959.

## Supplementary Materials

The following supporting information can be downloaded at: https://www.mdpi.com/article/10.3390/metabo12111136/s1. Figure S1: The neonatal breastmilk microbiota and the gut microbiota during the first week of life; Figure S2: Correlations of breastmilk microbiota and the global fecal metabolome in the first week of lactation; Figure S3: Mediation effect models of bacteria-related association between breastmilk sialylated oligosaccharides and the neonatal fecal metabolome; Figure S4: The important role of *Bacteroides* in milk–gut interaction and its association with the infant waist circumference at 1 year old; Table S1: Characteristics of the mothers and infants included in the study; Table S2: Concentrations of the stable isotope labeled internal standards; Table S3: Average relative abundance of breastmilk microbiota at the genus level during the first week of lactation; Table S4: Average relative abundance of newborn gut microbiota at the genus level during the first week of life; Table S5: General information of metabolites identified in newborn feces; Table S6: Partial correlation analysis of breastmilk microbiota and the neonatal fecal metabolome; Table S7: Partial correlation analysis between breastmilk sialylated oligosaccharides and newborn fecal metabolome; Table S8: Mediation effect model coefficients among breastmilk sialylated oligosaccharides-associated gut bacteria, breastmilk sialylated oligosaccharides, and the neonatal fecal metabolome; Table S9: Association of *Bacteroides* in breastmilk and newborn gut with fecal metabolites; Table S10: Settings of chromatographic gradient and valve switching time. References [54,55] are cited in the supplementary materials.
