# Supplementary material for: Effect of Breastmilk Microbiota and Sialylated Oligosaccharides on the Colonization of Infant Gut Microbial Community and Fecal Metabolome
Source: Metabolites. 2022 Nov 18;12(11):1136. doi: 10.3390/metabo12111136 (PMC9698434; doi:10.3390/metabo12111136)
Supplement: Supplementary file 1 [file metabolites-12-01136-s001.zip › Supplementary materials_edit_upload.pdf]

Supplementary Materials

Effect of Breastmilk Microbiota and Sialylated Oligosaccharides on the Colonization of Infant Gut Microbial Community and Fecal Metabolome

Juan Ding [1,†], Runze Ouyang [2,3,4,†], Sijia Zheng [2,3,4,†], Yanfeng Wang [2,3,4], Yan Huang [3], Xiao Ma [5], Yuxin Zou [6], Rong Chen [7], Zhihong Zhuo [8], Zhen Li [9], Qi Xin [10], Lina Zhou [2,3,4], Surong Mei [11], Jingyu Yan [2], Xin Lu [2,3,4], Zhigang Ren [12,*], Xinyu Liu [2,3,4,*] and Guowang Xu [2,3,4]

[1] Department of Quality Control, The First Affiliated Hospital of Zhengzhou University, Zhengzhou 450052, China
[2] CAS Key Laboratory of Separation Science for Analytical Chemistry, Dalian Institute of Chemical Physics, Chinese Academy of Sciences, Dalian 116023, China
[3] University of Chinese Academy of Sciences, Beijing 100049, China
[4] Liaoning Province Key Laboratory of Metabolomics, Dalian 116023, China
[5] Department of Nursing, The First Affiliated Hospital of Zhengzhou University, Zhengzhou 450052, China
[6] Liaocheng People's Hospital, Liaocheng 252000, China
[7] Dalian Municipal Women and Children's Medical Center (Group), Dalian 116011, China
[8] Department of Pediatric, The First Affiliated Hospital of Zhengzhou University, Zhengzhou 450052, China
[9] Department of Interventional Radiology, The First Affiliated Hospital of Zhengzhou University, Zhengzhou 450052, China
[10] Academy of Medical Sciences, Zhengzhou University, Zhengzhou 450052, China
[11] State Key Laboratory of Environment Health (Incubation), Key Laboratory of Environment and Health, Ministry of Education, Key Laboratory of Environment and Health (Wuhan), Ministry of Environmental Protection, School of Public Health, Tongji Medical College, Huazhong University of Science and Technology, Wuhan 430030, China
[12] Department of Infectious Diseases, The First Affiliated Hospital of Zhengzhou University, Zhengzhou 450052, China
* Correspondence: fccrenzg@zzu.edu.cn (Z.R.); liuxy2012@dicp.ac.cn (X.L.)
† These authors contributed equally to this work.

Supplementary Methods

Sialic acid and sialylated oligosaccharides quantification

Nontargeted neonatal fecal metabolomic analysis

Supplementary references

Supplementary Figures and Tables

Supplementary Methods

Sialic acid and sialylated oligosaccharides quantification

The online SPE-HILIC platform was established based on two High Performance Liquid Chromatography systems (HPLC, Shimazu, Kyoto, Japan) and a 2-position 6-port switching valve. One HPLC pump system was used as "cleanup pump" for purification and the other as "analysis pump" for analysis. The mobile phase of the cleanup pump consisted of ACN (A) and H_2O (B) with a flow rate of 0.2 mL/min. The mobile phase of the analysis pump consisted of ACN/H_2O/100 mM NH_4FA (pH = 3.2) (*v/v/v* = 8/1/1) (C) and H_2O/100 mM NH_4FA (pH = 3.2) (*v/v* = 9/1) (D) with a flow rate of 0.2 mL/min. The sample solution was injected when the 6-port valve was on the 1-2 position. Then the valve was switched to 6-1 position after 0.1 min to link the SPE cleanup column to the analytical HILIC column, by what the sample could pass through the two columns through using a gradient of ACN/H_2O (Table S10). At 5 min, the 6-port valve was switched to the initial position at which the two columns were at a "parallel" configuration.

The LTQ Orbitrap mass spectrometer (Thermo Fisher Scientific, Rockford, IL, USA) with electrospray ion (ESI) source was connected to the online SPE-HILIC platform for detection. The following conditions were applied: capillary temperature 325 ºC, source voltage -4.0 kV and capillary voltage -40 V for ESI- analysis. The mass scan range was set to 300 - 1000 Dalton. The resolution of the Orbitrap was set to 30,000.

TraceFinder software (version 3.2, Thermo Fisher Scientific, Rockford, IL, USA) was used for peak extraction. All the peak areas were corrected by that of SA-$^{13}C_6$. Then the absolute concentrations of SA, 3'-SL and 6'-SL were calculated by external standard method.

Nontargeted neonatal fecal metabolomic analysis

LC-MS based nontargeted metabolomic analysis was conducted on an Ultra Performance Liquid Chromatography (UPLC, Waters, Milford, MA, USA) -Q Exactive HF MS (Thermo Fisher Scientific,

Rockford, IL, USA) system with an ACQUITY UPLC BEH C8 column (Waters, 100 mm × 2.1 mm, 1.7 μm). In both ESI+ and ESI- modes, the mobile phases were water with 0.1% formic acid solution (A phase) and acetonitrile with 0.1% formic acid solution (B phase). The elution flow rate was 0.35 mL/min and the column temperature was 50 ºC. In ESI+ mode, the gradient started at 5% B, held for 1 min, and linearly increased to 55% B within 14 min, then arrived at 100% at 15 min and maintained for 3 min, finally returned to 5% B and held for 2 min for post equilibration. In ESI- mode, the gradient started at 2% B, held for 1 min, and linearly increased to 72% B within 13 min, then arrived at 100% at 14 min and maintained for 3min, finally returned to 5% B and held for 3 min for post equilibration. The MS conditions were set as the previous study [1].

Metabolite identification was performed by both OSI-SMMS database [2] and the mass bank of north America (MONA) database (https://mona.fiehnlab.ucdavis.edu/) according to accurate mass, retention time and MS/MS fragments. TraceFinder was used for peak extraction and then generated a peak list with the *m/z*, retention time and peak area of each sample. Inner standards were used for correcting the peak area.

Supplementary references

1. Wang, Q.; Su, B.; Dong, L.; Jiang, T.; Tan, Y.; Lu, X.; Liu, X.; Lin, X.; Xu, G. Liquid chromatography-mass spectrometry-based nontargeted metabolomics predicts prognosis of hepatocellular carcinoma after curative resection. *J. Proteome Res.* **2020**, *19*, 3533-3541.
2. Zhao, X.; Zeng, Z.; Chen, A.; Lu, X.; Zhao, C.; Hu, C.; Zhou, L.; Liu, X.; Wang, X.; Hou, X.; et al. Comprehensive strategy to construct in-house database for accurate and batch identification of small molecular metabolites. *Anal. Chem.* **2018**, *90*, 7635-7643.

Supplementary Figures

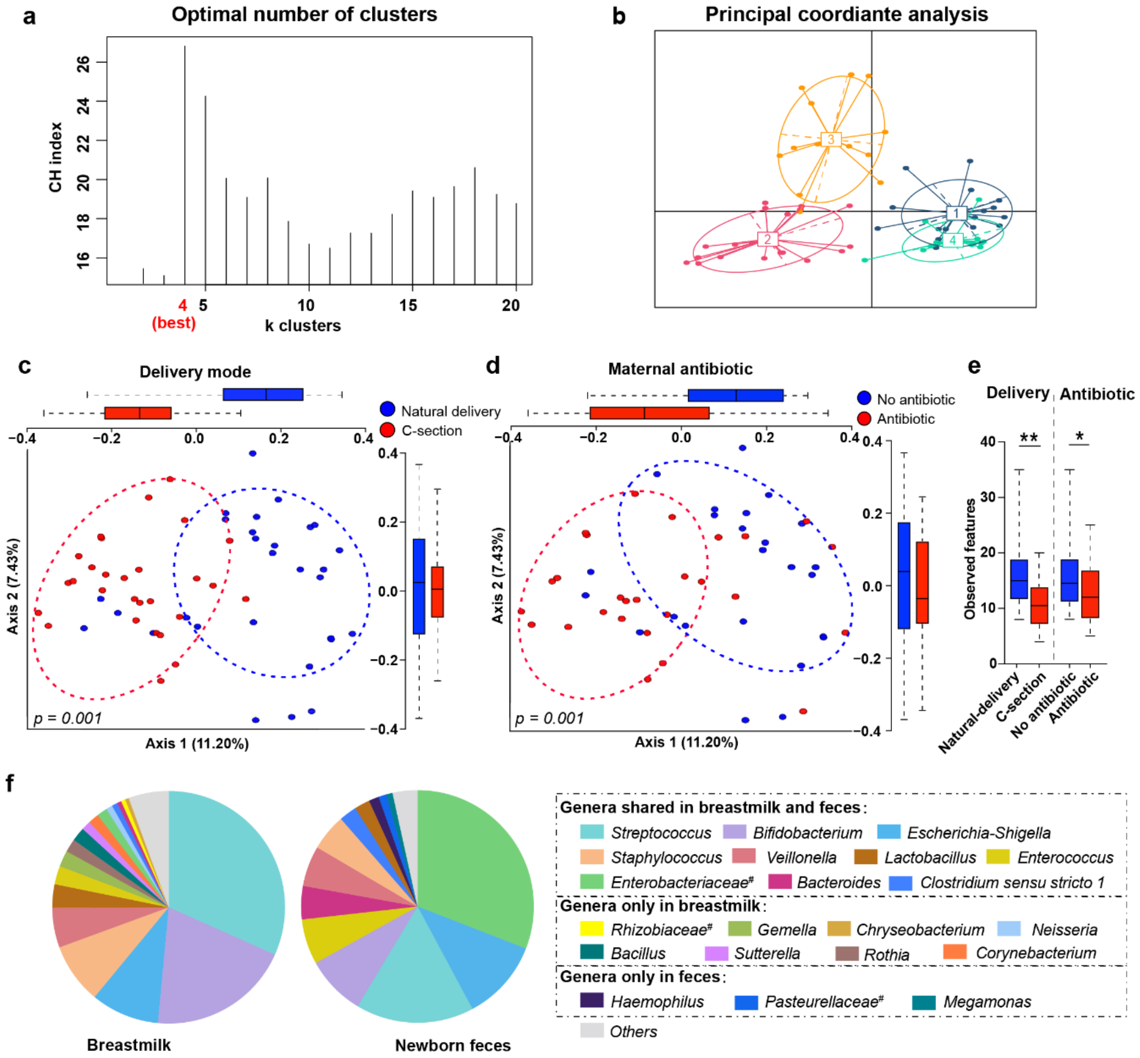

Figure S1. The neonatal breastmilk microbiota and the gut microbiota during the first week of life. (**a,b**) CH index indicates that the neonatal breastmilk microbiota could be better formed into four clusters. (**c**) PCoA plot of the gut microbiota in Natural-delivery and C-section infants based on Jaccard distance. (**d**) PCoA plot of the gut microbiota by maternal intake of antibiotics based on Jaccard distance. (**e**) Boxplots of alpha diversity calculated by observed features of the neonatal gut microbiota based on delivery mode and maternal antibiotic usage (Mann-Whitney U test, $*p < 0.05$). (**f**) Comparison of breastmilk microbiota and newborn gut microbiota composition at the genus level. Bacteria with relative abundance > 1% in either of breastmilk or feces are included in the pie chart. Pound sign ($^{\#}$) means unclassified bacteria at the genus level.

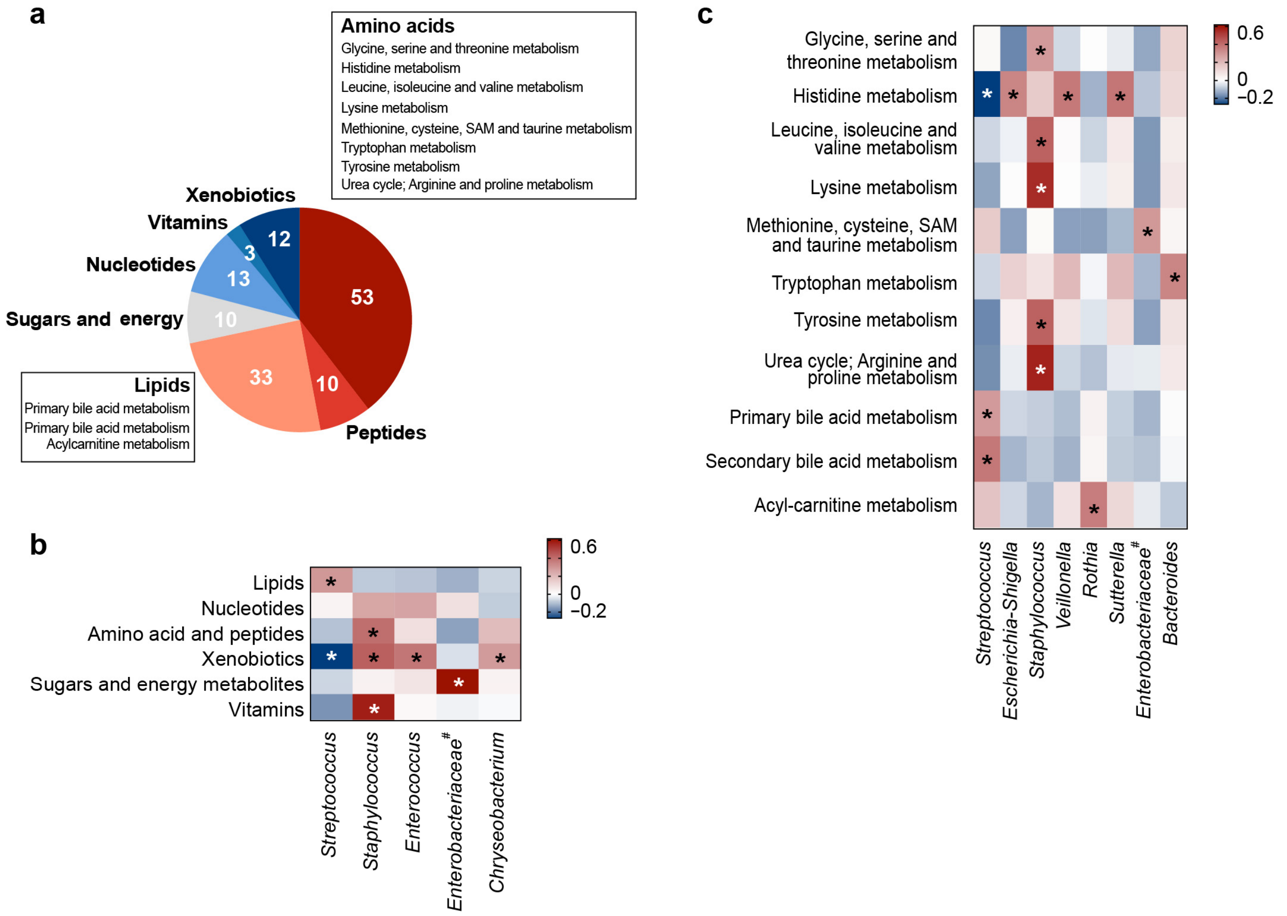

Figure S2. Correlations of breastmilk microbiota and the global fecal metabolome in the first week of lactation. (**a**) Number and category of neonatal fecal metabolites identified in the study. (**b**) Heatmap of partial correlation analysis between breastmilk microbiota and each category of fecal metabolite. (**c**) Heatmap of partial correlation analysis between breastmilk microbiota and metabolic pathway of neonatal fecal metabolome. Levels of metabolic pathway of neonatal fecal metabolome indicate the sum of the metabolites belonging to that pathway in this study. Asterisks (*) in heatmap mean significant correlation ($|R_1| > 0.2$ and $p < 0.05$). Pound sign (#) means unclassified bacteria at the genus level.

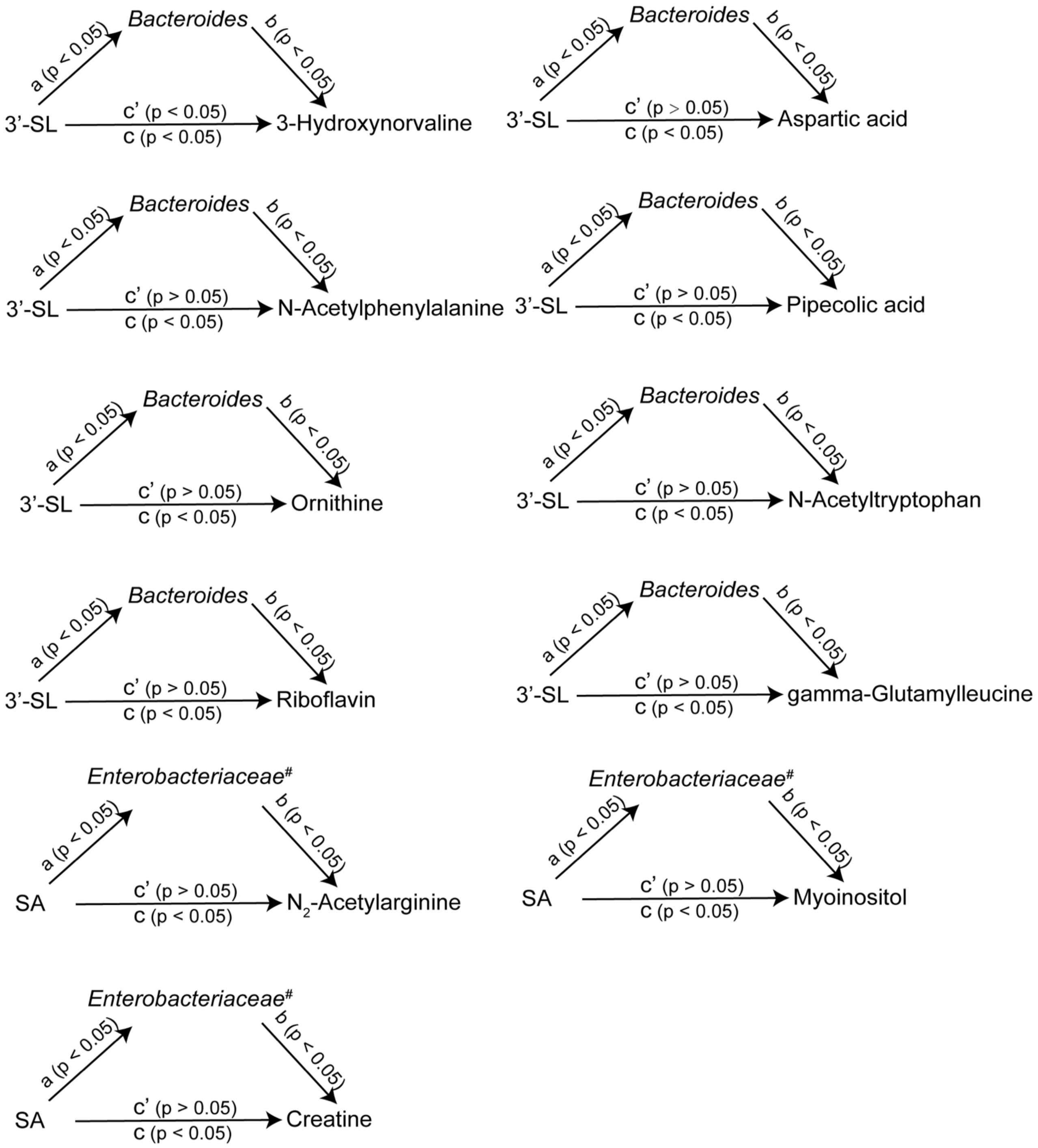

Figure S3. Mediation effect models of bacteria-related association between breastmilk sialylated oligosaccharides and the neonatal fecal metabolome. Pound sign (#) means unclassified bacteria at the genus level.

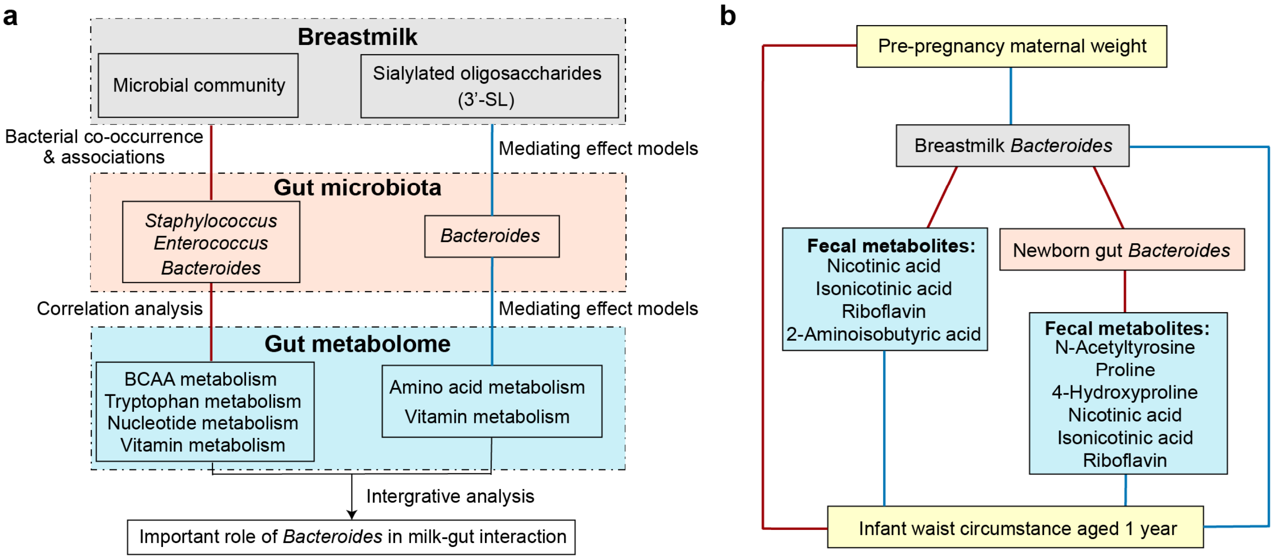

Figure S4. The important role of *Bacteroides* in milk-gut interaction and its association with the infant waist circumference at 1 year old. (**a**) Workflow of the comprehensive analysis of the effects of breastmilk microbiota and oligosaccharides on neonatal gut microbiota and fecal metabolome. 3'-SL: 3'-sialyllactose. (**b**) Frame of the correlation between *Bacteroides* and growth phenotype. Red lines indicate positive correlations and blue lines indicate negative correlations.

Supplementary Tables

Table S1. Characteristics of the mothers and infants included in the study. *(Provided in a separate Excel file)*

Table S2. Concentrations of the stable isotope labeled internal standards.

Internal standards	Abbreviation	Concentration (µg/mL)
Acetylcarnitine-d3	C2:0-d3	1
Decanoylcarnitine-d3	C10:0-d3	0.5
Palmitoylcarnitine-d3	C16:0-d3	0.5
Cholic acid-d4	CA-d4	2
Chenodeoxycholic acid-d4	CDCA-d4	1.6
Phenylalanine-d5	Phe-d5	6.7
Tryptophan-d5	Trp-d5	2.88

Table S3. Average relative abundance of breastmilk microbiota at the genus level during the first week of lactation.

Genera	Relative abundance (Mean)	Relative abundance (SD)	Prevalence
Streptococcus	31.69%	27.36%	100%
Bifidobacterium	19.80%	15.94%	100%
Escherichia-Shigella	9.50%	9.72%	100%
Staphylococcus	8.42%	15.05%	98%
Veillonella	5.54%	6.09%	98%
Lactobacillus	3.25%	6.92%	79%
Enterococcus	2.56%	6.96%	89%
Gemella	2.25%	6.56%	76%
Rothia	1.85%	4.23%	82%
Bacillus	1.93%	8.88%	52%
Sutterella	1.29%	2.01%	79%
Corynebacterium	1.50%	4.39%	68%
Enterobacteriaceae[#]	1.46%	2.89%	92%
Neisseria	0.89%	4.88%	58%
Clostridium sensu stricto 1	0.80%	1.02%	87%
Bacteroides	0.59%	1.86%	73%
Rhizobiaceae[#]	0.59%	1.43%	77%
Chryseobacterium	0.52%	3.37%	24%

Pound sign ([#]) means unclassified bacteria at the genus level. Genera with >1% average relative abundance in the neonatal breastmilk are shown.

Table S4. Average relative abundance of newborn gut microbiota at the genus level during the first week of life.

Genera	Relative abundance (Mean)	Relative abundance (SD)	Prevalence
Enterobacteriaceae#	30.91%	33.82%	86%
Escherichia-Shigella	11.26%	21.06%	65%
Streptococcus	16.43%	23.96%	95%
Bifidobacterium	8.33%	14.99%	74%
Enterococcus	6.28%	16.55%	61%
Bacteroides	4.64%	11.35%	40%
Veillonella	5.62%	14.08%	58%
Staphylococcus	5.10%	15.02%	89%
Clostridium sensu stricto 1	2.45%	7.34%	32%
Lactobacillus	2.15%	12.07%	33%
Haemophilus	1.41%	5.65%	44%
Pasteurellaceae#	1.11%	7.50%	9%
Megamonas	0.86%	3.91%	7%

Pound sign (#) means unclassified bacteria at the genus level. Genera with >1% average relative abundance in the neonatal gut are shown.

Table S5. General information of metabolites identified in newborn feces.

rt (min)	*m/z*	Metabolites	Category	Database
1.61	166.0861	Phenylalanine	Amino acid	OSI-SMMS
2.44	205.0971	Tryptophan	Amino acid	OSI-SMMS
0.83	182.0812	Tyrosine	Amino acid	OSI-SMMS
1.21	132.1017	Isoleucine	Amino acid	OSI-SMMS
1.12	132.1017	Leucine	Amino acid	OSI-SMMS
0.82	118.0861	Valine	Amino acid	OSI-SMMS
0.72	175.1189	Arginine	Amino acid	OSI-SMMS
0.74	132.0302	Aspartic acid	Amino acid	OSI-SMMS
0.76	148.0604	Glutamic acid	Amino acid	OSI-SMMS
0.75	76.0392	Glycine	Amino acid	MONA
0.71	154.0624	Histidine	Amino acid	OSI-SMMS
0.68	147.1128	Lysine	Amino acid	MONA
0.84	150.0583	Methionine	Amino acid	OSI-SMMS
0.77	116.0705	Proline	Amino acid	OSI-SMMS
0.75	106.0498	Serine	Amino acid	MONA
0.68	133.0971	Ornithine	Amino acid	OSI-SMMS
0.77	176.0917	Citrulline	Amino acid	OSI-SMMS
0.73	124.0074	Taurine	Amino acid	OSI-SMMS
0.73	118.0511	Threonine	Amino acid	OSI-SMMS
3.25	180.0654	Hippuric acid	Xenobiotics	MONA
0.77	160.0968	Isovalerylglycine	Amino acid	OSI-SMMS
1.01	217.1182	N_2-Acetylarginine	Amino acid	OSI-SMMS
0.78	175.1077	N_2-Acetylornithine	Amino acid	OSI-SMMS
0.80	189.1233	N_6-Acetyllysine	Amino acid	OSI-SMMS
1.11	188.0566	N-Acetylglutamic acid	Amino acid	OSI-SMMS
3.85	174.1124	N-Acetylleucine	Amino acid	MONA
2.83	192.0688	N-Acetylmethionine	Amino acid	MONA
3.97	206.0824	N-Acetylphenylalanine	Amino acid	OSI-SMMS
4.43	247.1075	N-Acetyltryptophan	Amino acid	MONA
2.89	222.0773	N-Acetyltyrosine	Amino acid	OSI-SMMS
0.74	146.0460	O-Acetylserine	Amino acid	OSI-SMMS
3.34	265.1180	Phenylacetylglutamine	Peptide	OSI-SMMS
1.83	203.1390	Alanylleucine	Peptide	MONA
2.68	281.1129	Aspartylphenylalanine	Peptide	MONA
2.71	261.1443	gamma-Glutamylleucine	Peptide	MONA
3.01	295.1285	Glutamylphenylalanine	Peptide	MONA
1.93	189.1233	Glycylleucine	Peptide	MONA
0.80	173.0921	Glycylproline	Peptide	OSI-SMMS
1.10	175.1077	Glycylvaline	Peptide	OSI-SMMS
0.82	229.1546	Leucylproline	Peptide	OSI-SMMS
1.90	233.1494	Threonylleucine	Peptide	OSI-SMMS
4.60	146.0600	Indole-3-carboxaldehyde	Amino acid	OSI-SMMS
4.93	176.0705	Indoleacetic acid	Amino acid	OSI-SMMS
2.79	190.0498	Kynurenic acid	Amino acid	MONA
0.68	112.0868	Histamine	Amino acid	OSI-SMMS
4.35	206.0811	Indolelactic acid	Amino acid	OSI-SMMS
0.78	139.0502	Urocanic acid	Amino acid	OSI-SMMS
0.75	134.0447	3-Hydroxynorvaline	Amino acid	OSI-SMMS

rt (min)	m/z	Metabolites	Category	Database
0.76	132.0655	4-Hydroxyproline	Amino acid	OSI-SMMS
1.02	138.0913	Tyramine	Amino acid	OSI-SMMS
1.38	190.1073	1-hydroxyhexanoylglycine	Lipid	OSI-SMMS
1.01	104.0528	2-Aminoisobutyric acid	Nucleotide	OSI-SMMS
0.81	118.0861	5-Aminopentanoic acid	Amino acid	OSI-SMMS
0.80	162.0760	Aminoadipic acid	Amino acid	OSI-SMMS
0.76	164.0917	Bicine	Xenobiotics	OSI-SMMS
0.76	132.0767	Creatine	Amino acid	OSI-SMMS
0.75	114.0662	Creatinine	Amino acid	OSI-SMMS
0.75	104.0705	gamma-Aminobutyric acid	Amino acid	MONA
0.75	120.0655	Homoserine	Amino acid	MONA
0.82	130.0863	Pipecolic acid	Amino acid	OSI-SMMS
1.02	130.0499	Pyroglutamic acid	Amino acid	MONA
2.26	122.0964	Phenylethylamine	Amino acid	MONA
0.75	241.0309	Cystine	Amino acid	OSI-SMMS
7.24	471.2417	CAS	Lipid	OSI-SMMS
6.04	464.3011	GCA	Lipid	OSI-SMMS
6.86	448.3064	GCDCA	Lipid	OSI-SMMS
6.28	528.2628	GCDCS	Lipid	OSI-SMMS
5.89	514.2841	TCA	Lipid	OSI-SMMS
6.76	407.2801	CA	Lipid	OSI-SMMS
7.84	391.2852	CDCA	Lipid	OSI-SMMS
6.16	405.2644	7-ketodeoxycholic acid	Lipid	OSI-SMMS
8.01	391.2852	DCA	Lipid	OSI-SMMS
8.73	377.2985	LCA	Lipid	OSI-SMMS
5.73	512.2680	GLCS	Lipid	OSI-SMMS
5.16	528.2630	GUDCS	Lipid	OSI-SMMS
0.77	209.0304	Glucaric acid	Sugar and energy metabolite	OSI-SMMS
0.78	260.0529	Glucosamine 6-phosphate	Sugar and energy metabolite	OSI-SMMS
0.76	193.0354	Glucuronic acid	Sugar and energy metabolite	OSI-SMMS
0.78	256.0594	N-Acetylglucosamine	Sugar and energy metabolite	OSI-SMMS
0.78	308.0987	N-Acetylneuraminic acid	Sugar and energy metabolite	OSI-SMMS
0.75	341.1086	Trehalose	Xenobiotic	OSI-SMMS
0.74	527.1370	Maltotriose	Sugar and energy metabolite	OSI-SMMS
0.75	162.1122	L-Carnitine	Lipid	OSI-SMMS
0.82	204.1230	Carnitine C2:0	Lipid	OSI-SMMS
3.18	246.1698	Carnitine C5:0	Lipid	OSI-SMMS
9.12	344.2792	Carnitine C12:0	Lipid	OSI-SMMS
8.73	396.3122	Carnitine C16:2	Lipid	OSI-SMMS
2.14	232.1542	Carnitine C4:0	Lipid	OSI-SMMS
7.60	316.2479	Carnitine C10:0	Lipid	OSI-SMMS
4.34	312.2165	Carnitine C10:2	Lipid	OSI-SMMS
8.39	342.2635	Carnitine C12:1	Lipid	OSI-SMMS
9.06	368.2791	Carnitine C14:0	Lipid	OSI-SMMS
9.94	370.2947	Carnitine C14:1	Lipid	OSI-SMMS
1.19	218.1386	Carnitine C3:0	Lipid	OSI-SMMS
4.28	260.1854	Carnitine C6:0	Lipid	OSI-SMMS
6.03	288.2166	Carnitine C8:0	Lipid	OSI-SMMS
6.98	314.2321	Carnitine C10:1	Lipid	OSI-SMMS

rt (min)	m/z	Metabolites	Category	Database

0.85	348.0687	3'-AMP	Nucleotide	OSI-SMMS
0.77	136.0618	Adenine	Nucleotide	OSI-SMMS
0.83	330.0581	Cyclic AMP	Nucleotide	MONA
0.76	112.0505	Cytosine	Nucleotide	OSI-SMMS
1.40	243.0974	Thymidine	Nucleotide	MONA
1.40	127.0502	Thymine	Nucleotide	MONA
0.83	111.0201	Uracil	Nucleotide	OSI-SMMS
1.06	243.0622	Uridine	Nucleotide	OSI-SMMS
0.81	166.0723	1-Methylguanine	Nucleotide	OSI-SMMS
1.11	183.0512	1-Methyluric acid	Xenobiotic	OSI-SMMS
0.82	137.0456	Hypoxanthine	Nucleotide	OSI-SMMS
1.01	151.0262	Oxypurinol	Xenobiotic	OSI-SMMS
0.77	179.0562	Paraxanthine	Xenobiotic	OSI-SMMS
0.81	169.0356	Uric acid	Nucleotide	OSI-SMMS
0.66	153.0393	Xanthine	Nucleotide	OSI-SMMS
1.03	147.0300	2-Hydroxypentanedioic acid	Lipid	OSI-SMMS
1.03	191.0198	Citric acid	Sugar and energy metabolite	OSI-SMMS
1.04	89.0245	Lactic acid	Sugar and energy metabolite	OSI-SMMS
0.82	133.0143	Malic acid	Sugar and energy metabolite	OSI-SMMS
0.69	188.1757	N_8-Acetylspermidine	Amino acid	OSI-SMMS
0.69	131.1291	N-Acetylputrescine	Amino acid	OSI-SMMS
0.91	87.0088	Pyruvic acid	Sugar and energy metabolite	OSI-SMMS
1.61	120.0807	Indoline	Xenobiotic	OSI-SMMS
1.01	165.0546	2-Hydroxycinnamic acid	Xenobiotic	OSI-SMMS
0.76	146.1175	4-Trimethylammoniobutanoic acid	Lipid	MONA
0.76	146.1175	Acetylcholine	Lipid	OSI-SMMS
0.73	104.1069	Choline	Lipid	OSI-SMMS
0.75	365.1051	Gentiobiose	Xenobiotic	MONA
0.75	179.0562	Myoinositol	Lipid	OSI-SMMS
0.98	124.0393	Isonicotinic acid	Xenobiotic	OSI-SMMS
0.88	124.0393	Nicotinic acid	Vitamin	OSI-SMMS
1.95	220.1179	Pantothenic acid	Vitamin	OSI-SMMS
1.01	136.0757	*p*-Octopamine	Xenobiotic	MONA
0.67	72.0807	Pyrrolidine	Xenobiotic	OSI-SMMS
0.67	146.1651	Spermidine	Amino acid	OSI-SMMS
3.34	377.1451	Riboflavin	Vitamin	MONA
4.35	130.0651	Indole-3-carbinol	Amino acid	MONA

Table S6. Partial correlation analysis of breastmilk microbiota and the neonatal fecal metabolome. *(Provided in a separate Excel file)*

Table S7. Partial correlation analysis between breastmilk sialylated oligosaccharides and newborn fecal metabolome.

Breastmilk sialylated oligosaccharides	Metabolites in newborn feces	R_1	*p*
SA	2-Hydroxypentanedioic acid	0.484	0.000
SA	Histamine	0.469	0.001
SA	Glycine	0.412	0.003
SA	Carnitine C4:0	0.387	0.005
SA	Oxypurinol	0.339	0.015
SA	Homoserine	0.339	0.015
SA	Tryptophan	0.332	0.017
SA	N-Acetylglutamic acid	0.322	0.021
SA	N_2-Acetylarginine	0.299	0.033
SA	Myoinositol	-0.286	0.042
SA	Creatine	0.278	0.048
3'-SL	3-Hydroxynorvaline	-0.431	0.002
3'-SL	Aspartic acid	-0.329	0.018
3'-SL	N-Acetylphenylalanine	-0.329	0.019
3'-SL	Pipecolic acid	-0.323	0.021
3'-SL	Ornithine	-0.317	0.023
3'-SL	N-Acetyltryptophan	-0.310	0.027
3'-SL	Riboflavin	-0.288	0.041
3'-SL	gamma-Glutamylleucine	-0.285	0.043
6'-SL	N-Acetylputrescine	0.391	0.005
6'-SL	Glucaric acid	-0.318	0.023
6'-SL	Thymine	0.309	0.027
6'-SL	Thymidine	0.298	0.033
6'-SL	Glucuronic acid	-0.298	0.034

Table S8. Mediation effect model coefficients among breastmilk sialylated oligosaccharides-associated gut bacteria, breastmilk sialylated oligosaccharides and the neonatal fecal metabolome. *(Provided in a separate Excel file)*

Table S9. Association of *Bacteroides* in breastmilk and newborn gut with fecal metabolites.

Bacteria	Fecal metabolites	B			*p*
		Mean	Lower limit	Upper limit	
Breastmilk *Bacteroides*	Isonicotinic acid	5.61E-02	1.03E-01	9.29E-03	0.020
	Nicotinic acid	3.02E-02	5.30E-02	7.49E-03	0.010
	2-Aminoisobutyric acid	1.64E-03	3.24E-03	3.14E-05	0.046
	Riboflavin	6.67E-04	1.25E-03	8.86E-05	0.025
Newborn gut *Bacteroides*	Proline	9.78E-02	1.89E-01	6.58E-03	0.036
	Isonicotinic acid	9.98E-03	1.78E-02	2.15E-03	0.014
	Nicotinic acid	5.17E-03	8.97E-03	1.36E-03	0.009
	N-Acetyltyrosine	2.49E-03	4.39E-03	5.89E-04	0.011
	4-Hydroxyproline	8.42E-04	1.50E-03	1.88E-04	0.013

Blue indicates significant associations of breastmilk *Bacteroides* with fecal metabolites; Red indicates significant associations of newborn gut *Bacteroides* with fecal metabolites.

Table S10. Settings of chromatographic gradient and valve switching time.

Cleanup pump

Time (min)	A%[a]	B%[a]
0	80	20
10	80	20
30	40	60
30.1	80	20
47	80	20

Analysis pump

Time (min)	C%[a]	D%[a]
0	100	0
6	100	0
36	44	56
38	44	56
38.1	100	0
47	100	0

Valve cut time

Time (min)	Valve position
0	1-2
0.1	6-1
5	1-2

a. Mobile phase: A, ACN; B, H_2O; C, ACN/H_2O/100 mM NH_4FA (pH=3.2) (*v/v/v* = 8/1/1); D, H_2O/100 mM NH_4FA (*v/v* = 9/1).
